# Multiscale and multiresolution modeling of shales and their flow and morphological properties

**DOI:** 10.1038/srep16373

**Published:** 2015-11-12

**Authors:** Pejman Tahmasebi, Farzam Javadpour, Muhammad Sahimi

**Affiliations:** 1 Bureau of Economic Geology, Jackson School of Geosciences, The University of Texas at Austin, 78713, Austin, TX, USA; 2 Mork Family Department of Chemical Engineering and Materials Science, University of Southern California, Los Angeles, California 90089-1211 USA

## Abstract

The need for more accessible energy resources makes shale formations increasingly important. Characterization of such low-permeability formations is complicated, due to the presence of multiscale features, and defies conventional methods. High-quality 3D imaging may be an ultimate solution for revealing the complexities of such porous media, but acquiring them is costly and time consuming. High-quality 2D images, on the other hand, are widely available. A novel three-step, multiscale, multiresolution reconstruction method is presented that directly uses 2D images in order to develop 3D models of shales. It uses a high-resolution 2D image representing the small-scale features to reproduce the nanopores and their network, a large scale, low-resolution 2D image to create the larger-scale characteristics, and generates stochastic realizations of the porous formation. The method is used to develop a model for a shale system for which the full 3D image is available and its properties can be computed. The predictions of the reconstructed models are in excellent agreement with the data. The method is, however, quite general and can be used for reconstructing models of other important heterogeneous materials and media. Two biological examples and from materials science are also reconstructed to demonstrate the generality of the method.

Due to the substantial amount of natural gas that they store, shales have emerged as one of the most promising current and future energy resources. Shale gas can be dissolved in kerogen, be adsorbed on clay and kerogen, and/or appear as free gas in pores and natural fractures^1^,^2^. Most of the gas production in such porous formations occurs in the source area because, owing to the low permeability of the formations^3^, the gas does not migrate significantly.

Unlike conventional large-scale reservoirs that have been characterized through countless methodologies^4^, shale formations have not yet undergone advanced characterization, and much remains to be done. A most important obstacle to characterization and modeling of shales, which up to now has seemed insurmountable, is their multiscale structure that defies the conventional techniques of characterization. Pores of all sizes, ranging from nano to micro exist in shales^5^,^6^, which play a vital role in fluid flow^7^,^8^. The intrinsic small scale and spatial variability of such pores require a very large number of samples for direct characterization of shales that, if available, will make it possible to better understand the depositional environment, mineralogy, maturation, thermal condition, total organic carbon, strain/stress properties, porosity, and permeability of shales. But, shales are also large-scale porous media and, therefore, the spatial variations of their properties also occur not only at very large scales. Thus, there are several distinct and disparate length scales that are relevant to modeling and characterization of shales.

Two- or three-dimensional (3D) imaging of shale samples have become an important tool for their characterization, because they reveal very useful information about their pore network and their spatial connectivity, and provide a reliable platform for their characterization. In addition, some of the aforementioned key morphological and flow properties of shales, such as the TOC, mineralogy, porosity, permeability, and other physical properties can be extracted using such images^9^. As a result, the acquisition of high-resolution 2D and 3D images through the use of focused ion-beam scanning electron microscopes^10^ (FIB-SEM) is viewed an essential part of such studies. While high-resolution 2D images can be obtained with relative ease, acquisition of 3D images that are necessary for precise characterization is costly and time consuming. Furthermore, some information, such as pore spots, can be lost during layer milling^10^. Thus, obtaining high-quality 3D images has become an outstanding challenge.

Given the availability of 2D images, reconstructing a representative 3D sample using such images would be very efficient, cost-effective and beneficial. Acquiring and processing a 2D SEM image takes minutes to hours and costs hundreds of dollars, whereas processing a 3D FIB-SEM image takes days and costs tens to hundreds of thousands of dollars. Another disadvantage of the FIB-SEM images is their tiny size—tens of nanometers edge size—compared with the 2D SEM images that can be as large as hundreds of micrometers in edge. Because of the labor, cost, and limited size, if one can develop a technique to use only 2D images, then there would be no need to fully scan a sample in 3D when a few 2D images or even one single, relatively large 2D image can convey even more information about the heterogeneity and morphology of the sample.

Some efforts in this direction have been made. Slatt, O’Brien and co-workers^11^,^12^,^13^ proposed a method for using 2D SEM images of shales, obtained from carefully-broken clean 2D shale surface of a cubic sample. For each surface several separate viewing areas are selected at random and photographed, and are then used to estimate the sample’s various properties. Several magnifications of the images are used to estimate the properties at different “scales,” and in particular the “area” porosity, i.e. the porosity based on surface not volume of a sample. The method can yield important insights into the structure of shales at small scales.

Several statistical methods are also available that extract the important statistics/properties, and then generate stochastically many realizations of the formation^14^,^15^,^16^,^17^. Typically, such methods are based on an optimization technique, such as simulated annealing, by which one minimizes the differences between the inferred statistical properties from the initial dataset and those in the simulated model. The extracted statistics using such methods are, however, poor and fail to reproduce a high-quality model. In another group that is process based, one tries to mimic the real physical process to develop a model^18^,^19^,^20^. Such methods are not computationally feasible. They also require considerable calibrations due to the extreme heterogeneity and variability of shale formations, and do not represent a general approach as each method is developed for a specific type of porous media. In addition, most of the current methods are based on some low-order statistical descriptors that cannot reproduce the complex structures that are abundant in shales. Moreover, they are unable to use directly *qualitative* information, such as insight into the type of structure that a given formation may be expected to have. Consequently, all the necessary details of the physical process and morphological information must first be “*extracted*” and then “*translated*” numerically, and even then only some of the information can be used effectively. Statistically, reproducing long-range connected features that are important to flow and transport is not possible using a limited number of data points. Generating such features requires processing a large amount of data over extended length scales (i.e., higher-order statistics) at the same time. Providing such “*large data sets”* is not, however, feasible in the earth sciences and, in particular, for modeling of large-scale porous media.

All the aforementioned shortcomings can be addressed using a conceptual data set called a digital image, which represents auxiliary information that brings in the effect of the physical process and extra supplementary data that can be used in the framework of high-order statistics, in order to develop an accurate model. The digital image can simply be the result of process-based processes that have given rise to the formation, or a SEM image. Once the image is available, an important and heretofore unexplored approach to modeling of shales can be developed, which is based on *reconstruction*: Given a certain amount of data, one tries to reconstruct a model for shales that not only honors the data, but also provides accurate predictions for those properties for which there are no data or, if such data are available, they are not used in the reconstruction.

The aim of this paper is to propose a new, accurate, and efficient reconstruction method for modeling multiscale heterogeneous media using 2D SEM images. The method reconstructs the corresponding 3D model for a shale formation using the 2D images. Unlike previous methods, the available dataset is used directly in the proposed method, thereby eliminating the need for data extraction. We show how to avoid taking a large number of costly and time-demanding high-resolution 2D images and, instead, use a combination of just a few low- and high-resolutions images to rapidly reconstruct models of 3D shales. To our knowledge, the present work is the first study on shale reconstruction that reduces the cost and time tremendously. The results of this study can be used for accurate modeling of fluid flow and transport in such complex large-scale porous media. Most importantly, as we demonstrate in the supplementary information, the method is general and may be used to reconstruct 3D models of a wide variety of heterogeneous materials and media

## Results and Discussion

**Figure 1:**
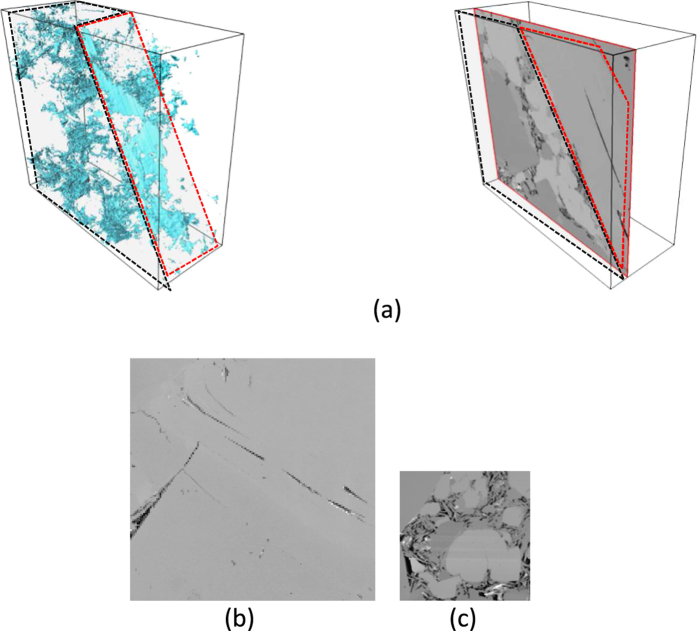
(**a**) 3D view the pore sizes at two different scales, indicated by dashed red and black lines (left image), and a cross section of the sample showing the large- and nano-scale pores (right image). (**b**) The digital image used for reconstruction of the large-scale pores, and (**c**) the selected nanoscale digital image. The size of the systems in (**a**–**c**) is 22 × 19 × 8.5 (μm^3^), 22 × 19 (μm^2^) and 10 × 10 (μm^2^), respectively.

To test the methodology, a highly complex sample is selected. The 3D image of the sample is available; see Fig. 1(a). Thus, we first use the 3D image of the sample to compute its permeability and porosity. After the 3D model of the sample is reconstructed based on 2D images, a comprehensive comparison of its flow and physical parameters with those of the original 3D sample is presented.

### Reconstructed model

To apply the method to a difficult but also realistic problem, a minimum amount of information for the sample, namely, two images, one high resolution and a second one with low resolution are extracted from the 3D image. Figure 1(b) depicts the large-scale pores that are the main flow paths in the shale, representing the low-resolution image. Figure 1(c) represents the high-resolution image that exhibits nanoscale pores, distributed between the large channels, which play an important role in connecting the large-scale pores.

**Figure 2:**
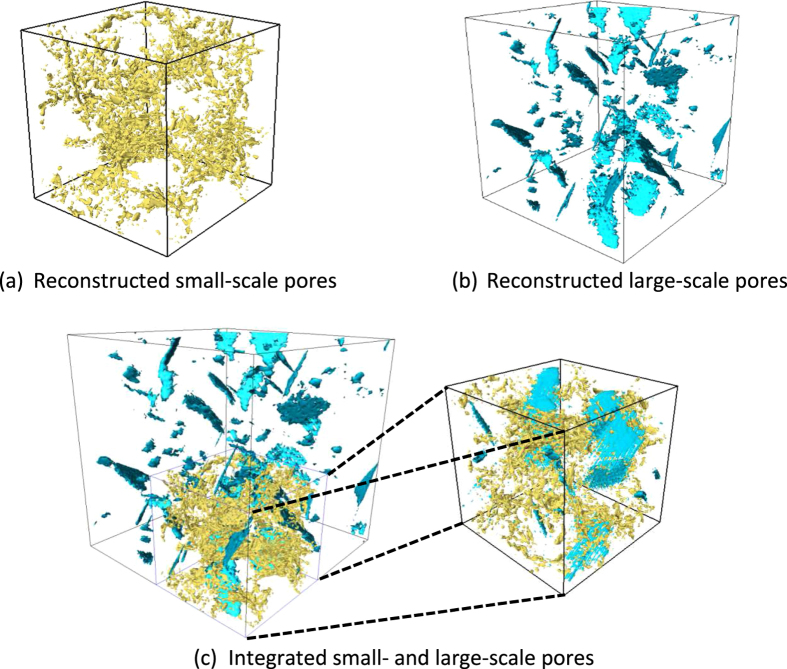
Three-dimensional view of the reconstructed (**a**) small- and (**b**) large-scale pores. (**c**) The integrated model. For the sake of illustration, only a quarter of the large-scale grid is filled and shown. The size of the small and large 3D models is 22 × 22 × 22 (μm^3^) and 10 × 10 × 10 (μm^3^), respectively.

The nanoscale shale sample contains two very different pore structures that are at two separate scales. Therefore, the proposed multiscale approach is implemented for the sample as follow: First, the representative image shown in Fig. 1(c) that depicts the fine-scale nanopores is used to stochastically reconstruct several realizations using the method described below. One reconstructed realization is presented in Fig. 2(a). Then, using the low-resolution digital image in Fig. 1(b), the large-scale pores are reconstructed; see Fig. 2(b). Finally, the reconstructed models for the two disparate scales are superimposed to achieve a single model; see Fig. 2(c). It is clear that the large-scale model reproduces the main features of the pore space at the corresponding scales, while the paths are connected through the nanopores. We now compare the properties of the reconstructed models with those of the original 3D sample.

### The auto-correlation function and probability distribution

**Figure 3:**
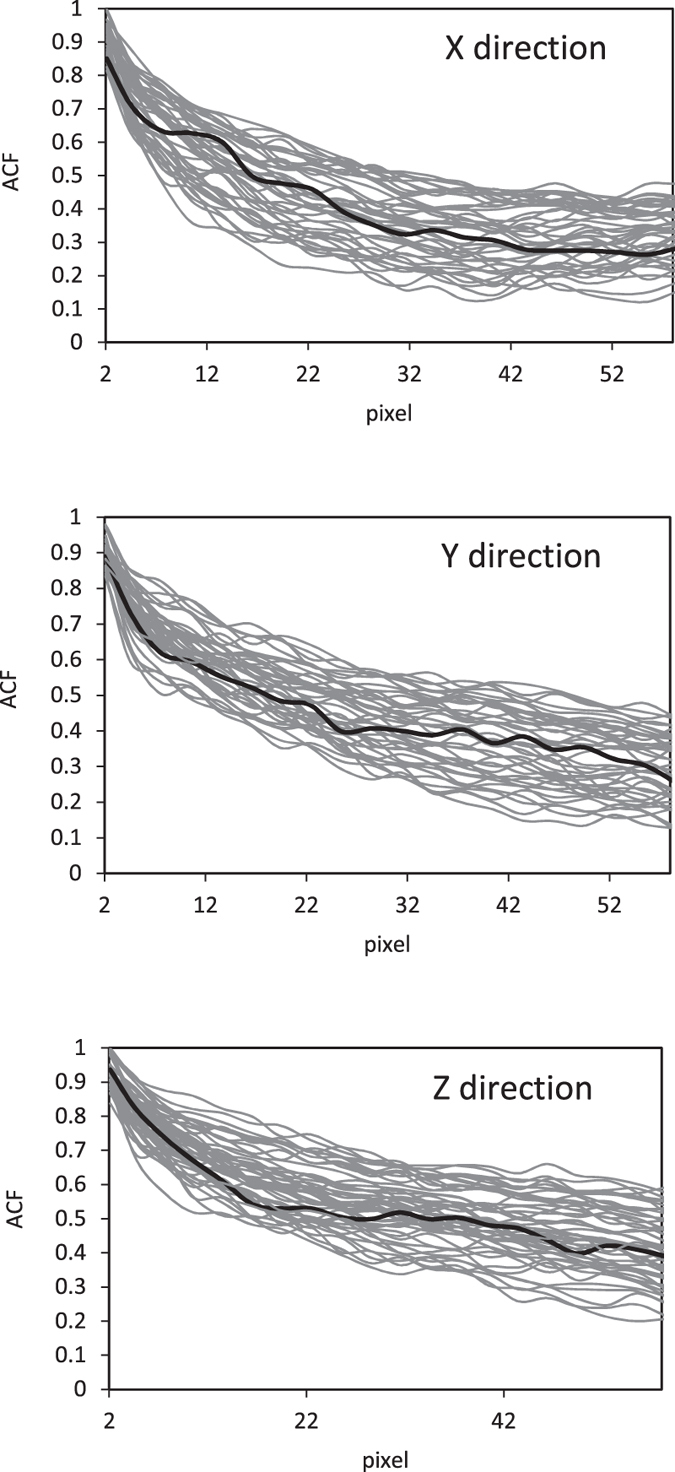
Comparison of the auto-correlation functions of the original (thick black) and the realizations of the reconstructed models (gray).

The pore-pore autocorrelation function is defined by


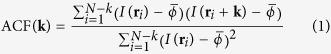


where *r*_*i*_ indicates the spatial location within the model, and 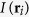 is an indicator, such that 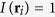 if *r*_*i*_ is in the pore space, and 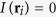 otherwise. 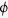 is the porosity. The auto-correlation functions of the original sample and the reconstructed models were computed for three orthogonal directions and shown in Fig. 3. Clearly, the two sets of results agree. The accuracy of the auto-correlation function is not, however, sufficient for verifying the accuracy of the reconstructed models.

**Figure 4:**
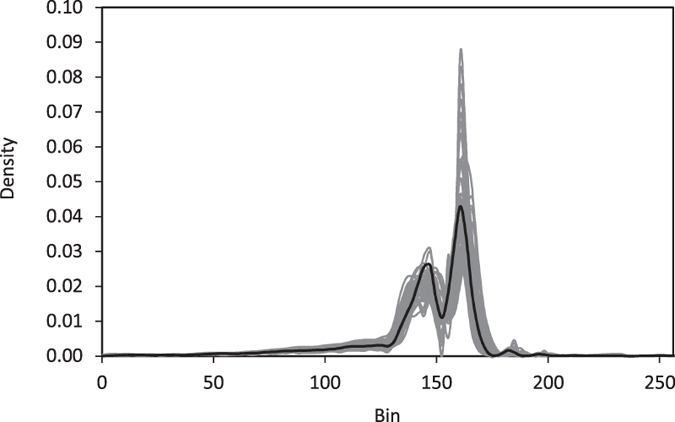
Comparison of the probability distributions in the realizations of the reconstructed models (gray) with that of its original 2D digital images (black). The density represents the relative likelihood of the model to take a given value in a bin.

Using the probability distribution of the pixels as a separate constraint improves the reproduction of the nano- and micropores. Naturally, since we use the cross-correlation simulation (CCSIM) algorithm to begin the reconstruction (as described below) that uses a cross-correlation function representing higher-order statistics of the formation, we expect the probability distribution to be reproduced. However, the exact distribution of the original 3D sample might not be reproduced due to utilizing one 2D image to reconstruct the 3D formation. Thus, the probability distributions of several realizations are compared with that of the original 3D sample; see Fig. 4. The complex multimodal probability distributions are well reproduced.

### Multiple-point connectivity probability

**Figure 5:**
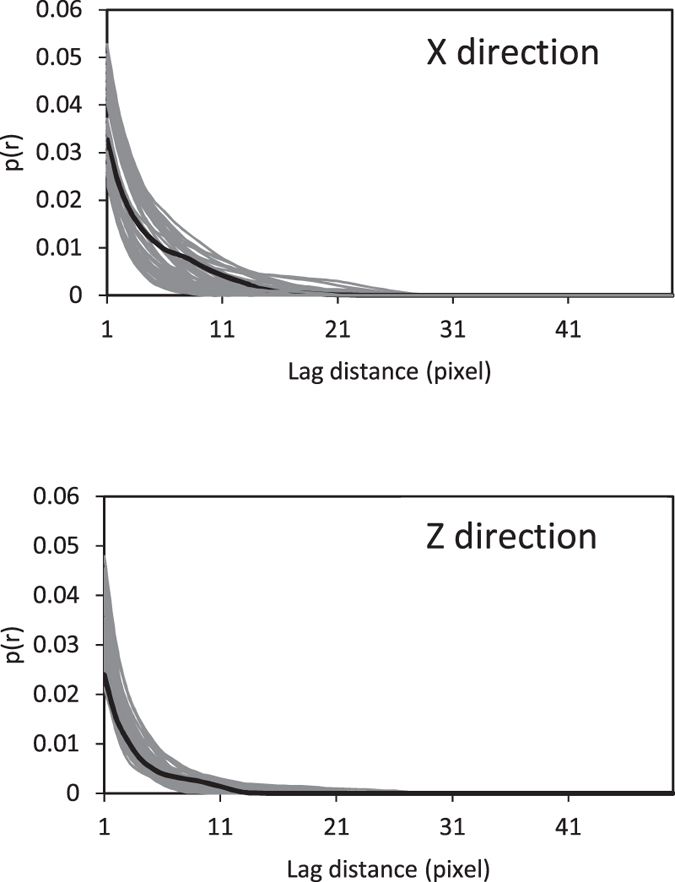
Comparison of the multiple-point connectivity probability in the original 3D sample (thick black) and the realizations of the reconstructed models (gray) in two directions.

The comparisons so far involved lower-order statistical properties that only account for a probability/occurrence of the individual points. Large-scale connectivity is one of the most important properties that should be reproduced, as it controls flow in porous media^4^. In particular, multiple-point connectivity is^21^ concerned with the probability p(**r**; *m*) of having a sequence of *m* points connected in a given phase of a multiphase material (e.g. pore and matrix) in a direction **r**. It is defined by


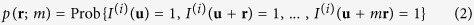


where 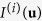 is the indicator function of phase *i* defined earlier. *p*(**r**;m) accounts for the global connectivity of a system and represents a strict test of the reconstruction method’s accuracy. The results are shown in Fig. 5. The agreement between the computed probabilities of the original 3D sample and the generated stochastic models is excellent.

### Effective permeability

Despite the high accuracy of the reproduced statistical properties described so far, they are still insufficient for asserting that the reconstructed model is accurate, because it is possible for two media to have similar statistics for their morphological properties, but very different flow characteristics. Therefore, we present a comparison between the flow properties of the original samples and those of the generated 3D models.

**Figure 6:**
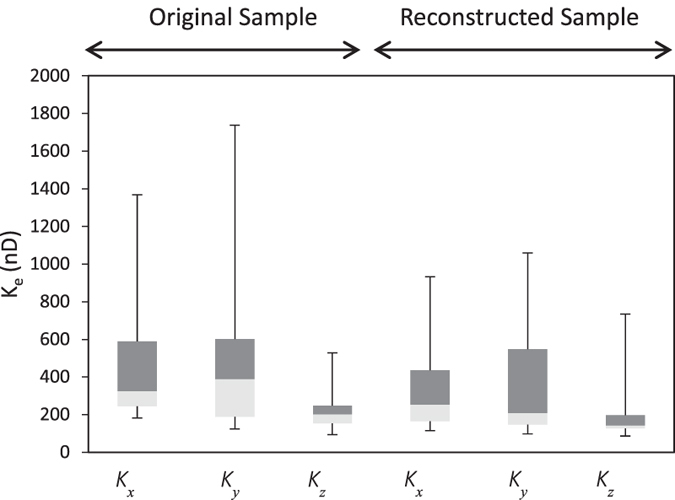
Boxplot of the directional effective permeabilities of the original and reconstructed samples, showing their possible ranges.

The original 3D image contains 4000 × 4000 × 1800 voxels. The effective permeability K_e_ is a measure of the ability of a pore space for fluid flow. Due to the extreme spatial heterogeneity and anisotropy associated with the samples, a single effective permeability cannot realistically characterize the flow property of a large shale sample. Therefore, the directional effective permeabilities were calculated for three orthogonal directions and regions within the 3D sample, using commercial software Avizo®. To obtain estimates of the range of the possible variations of K_e_, several smaller pieces of the 3D images were used to compute the effective permeabilities. Avizo® was also used for the permeability calculations of the reconstructed samples. In all cases, the Stokes’ equation,


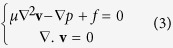


was solved numerically, assuming that the fluid is incompressible and Newtonian, and the flow is at steady state and in the laminar regime. Here, *p* is the pressure of the fluid, **v** is its velocity, and 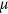 is its dynamic viscosity. The no-slip condition at pore-fluid interfaces was assumed. A pressure difference of 3 × 10^4^ Pa (upstream = 13 × 10^4^ Pa, and downstream = 10 × 10^4^ Pa) and a fluid viscosity of 0.001 Pa.s were used in the calculations. The results are presented in Fig. 6, indicating very good agreement with results of the original 3D samples.

## Conclusions

Realistic models of complex porous media cannot be developed based on low-order statistics alone. In particular, if they represent multiscale systems with distinct, disparate and relevant length scales and heterogeneities at all the scales, one needs an approach that utilizes higher-order statistics and information at all the relevant scales by a computationally-affordable method. Shale formations represent an important class of such porous media. This paper presented a multiscale, multiresolution method for reconstructing models of highly complex porous media, and demonstrated its accuracy for a shale sample. The method combines several elements: an iterative 3D reconstruction, a probability distribution matching, a multiresolution algorithm, and a multiscale approach. The iterative algorithm removes the possible artifacts during the initial reconstruction process. The probability distribution matching prevents artificial smoothness and guarantees an accurate reproduction of the complex multimodal shale distribution. The intrinsic multiscale pore structures in shale formations are reproduced using two efficient methods, namely, multiscale and multiresolution imaging. Altogether, the new technique presented in this paper is able to accurately reproduce the complex pore features in shale samples. It can be used as a tool for reducing the cost and time in the studies of shale and related structure for which high-quality 3D images are required.

Multiscale materials and media are abundant in nature and occur widely in other fields, such as biology, nanotechnology, and materials science. The method presented here can also be used to model such materials and media as well. Three examples to which the proposed method was applied are provided in the Supplementary Information. Therefore, the method is not restricted to the shales considered in this paper, and can provide models for other important heterogeneous media and materials for which one has a few high- and low-resolution 2D images and the aim is to generate 3D models for them.

## Methodology

The proposed methodology consists of three steps. Each step relies on the model developed in the previous steps, and improves it.

### Step 1

**Figure 7:**
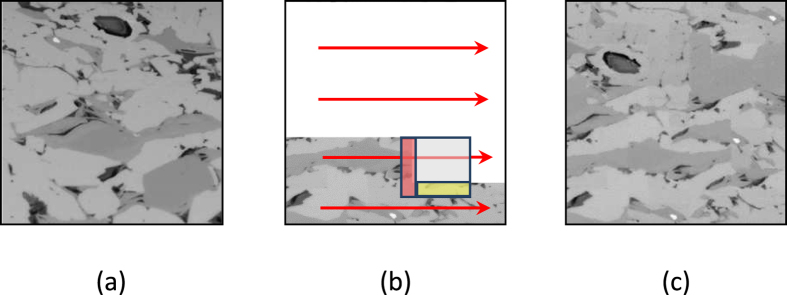
(**a**) The digital image. (**b**) The procedure for overlap consideration (narrow red and yellow bands) for identifying the next candidate pattern in the gray template, and the raster path (red arrows). (**c**) The reconstructed model. The size of the image is 6 × 6 μm^2^.

This step utilizes the cross correlation-based simulation (CCSIM) method, which belongs to a class of stochastic models that aims to produce an ensemble of equi-probable realizations^22^ of disordered media, such as porous formations. The method is cast on a computationally fast cross-correlation formulation that allows achieving efficient and accurate modeling in a very short time. First, a representative 2D image is selected as the digital image; see Fig. 7(a). The image must contain most of the expected features, including the connectivity and distribution of the pores, the porosity, and other important factor, and is set as the first layer (external surface) at the bottom of the 3D model, represented by a 3D cubic computational grid **G**. Then, five frames that, together with the digital image form the six orthogonal outside surface of the model are, reconstructed based on the CCSIM algorithm (see below). The reconstruction is *conditional*, i.e. each frame is reconstructed subject to honoring the data at the interface between itself and the already existing frames. Thus, the first reconstructed layer honors the data at its interface with the digital image, the next frame honors the data at its interface with any of the existing frames (including the digital image, if applicable), and so on.

As shown in Fig. 7, the CCSIM algorithm reconstructs the system conditionally and plane by plane, after doing so for the first five external frames or planes. The 2D computational grid (plane) is partitioned into small blocks called templates **T**, while the data event at position **u** in **T** is denoted by **D**_*T*_(**u**), so named to signify the fact that it can change during the reconstruction. Every two neighboring templates share an overlap region. Then, the other four frames (i.e. left, right, front, and back) are reconstructed, starting from a corner of the grid **G** and proceeding along a 1D raster path, as shown in Fig. 7. At each step, to preserve the continuity between the patterns in the neighboring blocks, an overlap region is considered and its similarity to the *entir*e digital image is calculated using the following cross-correlation function:


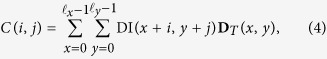


where 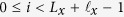 and 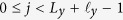, and 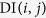
represents the location at point 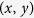 of the digital image that is used to reconstruct a target system of size 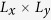, with 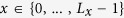 and 
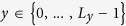
. The **overlap** region 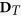 of size 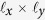 is used to match the pattern in the selected block with the digital image. Any realization of an overlap region with a cross-correlation function larger than a threshold is an acceptable pattern. Finally, one of the acceptable patterns in the ensemble of the realizations is selected at random and inserted in the current data event **D**_*T*_ in the computational grid **G**.

Since the reconstruction of each layer (plane) is conditional, optimal locations of the hard data—those that must be honored *exactly*—in the already reconstructed layers must be specified. Each layer is conditioned to the hard data in the immediately reconstructed layer before it, and to the data at its interfaces with the four external frames (reconstructed at the beginning) that it touches. The hard data must be selected from the immediately-reconstructed layer. To do so, we use the Shannon entropy *S*: For each template of the immediately-reconstructed layer the entropy *S* is computed. Since a more heterogeneous template has higher entropy, the template is split recursively if *S* is high. The splitting continues until the right template size—one that contains low entropy—is generated. Next, a fraction of the hard data in the final template with the lowest entropy is selected, and the next layer is reconstructed. Finally, all the generated layers are stacked together to build the 3D model. Clearly, one can generate as many realizations as needed. Note that different digital images can be selected on the basis of the sample’s complexity and heterogeneity. For example, various digital images for illustrating the vertical, horizontal, and lateral directions can be utilized.

### Step 2

**Figure 8:**
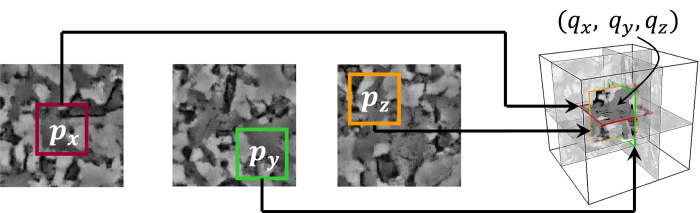
Iterative 3D refining: all the points are visited in the initial 3D output (far right). Then, three perpendicular data events 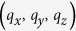 are extracted at each visiting point. Next, the best candidate for each data event is identified in its corresponding digital image using the cross-correlation function, defined by Eq. (4). Every digital image in this figure has a size of 150 × 150 pixels (5 × 5 μm^2^). Finally, the proposed probability distribution matching is implemented to modify the visiting point in the 3D model.

The complete continuity of the reconstructed model is sometimes not achievable by Step 1, and the generated realizations may contain some artifacts. Thus, the model in Step 1 is further improved using an iterative scheme to minimize the discontinuities and reproduce patterns that are even more similar to those in the digital image, while preserving their continuity. To this end, the 3D model reconstructed in Step 1 is further refined voxel by voxel. A voxel in the 3D model and three orthogonal patterns 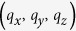 around it are selected from the 3D model; see Fig. 8. Then, the cross-correlation functions between the patterns and the 2D digital images are computed and, in each plane, the closest patterns 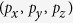, i.e. those for which the cross-correlation functions are the largest, are selected. An average of the selected patterns is assigned to the selected voxel. The averaging might, however, produce artifacts resulting in a 3D model that may be too smooth and not suitable for computing the flow properties, because the low- and high-permeability areas have been averaged out. We will come back to this issue shortly.

### Step 3

As mentioned, shale formations exhibit a sharp bimodal distribution of pore sizes^9^,^23^. Nano- and microscale pore structures are very common in shales, and *both* features should appear in a realistic model for them. Indeed, it has been demonstrated through laboratory experiments that the nanopores along microspores’ networks control gas transport^1^,^7^,^24^, as they provide the connection between them. Thus, their presence, representing an interconnected pore network, is very important. The large-scale structures make up the main pore network, even though they are connected through the nanopores. Since having a high-quality 2D image of a *large* sample is not feasible due to its high cost and time-demanding processing, it is preferable to take two images with different resolutions: (a) one *large 2D low-resolution* image for capturing the microscale structure, which is easily obtained, and (b) one or a few *small 2D high-resolution* images for taking into account the structure of the nanopores’ network. The two sets of images are viewed as complementary datasets for revealing the main structures within a shale sample. To address these issues, three complementary methods are presented, and utilized: a probability distribution matching, and a multiresolution approach, both combined with a multiscale method.

#### Probability distribution matching

Reproducing the multimodal pore structure is an important issue, not only in shales, but also in most earth science problems. For example, most large-scale porous media contain a broad permeability distribution. In such porous media the emphasis is on the high-permeable zones. Due the overall low permeability of shales, however, all the small-scale features are also important and must be included in the final model.

**Figure 9:**
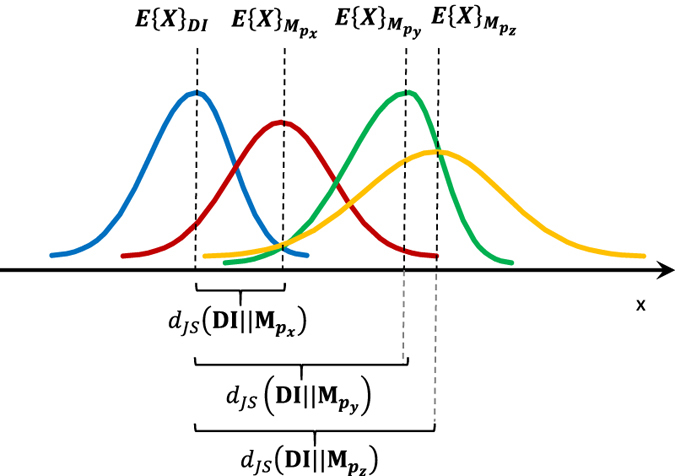
Illustrating the application of pattern selection and probability distribution matching in which the difference between each set of patterns 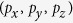 and the digital image is quantified using the Jensen-Shannon method. Eventually, a pattern with the minimum distance is selected (red distribution). *E* denotes the expected value.

The probability distribution matching provides a method to control the patterns in the 3D model that was produced by Steps 1 and 2, and helps one to select one of the candidate patterns more efficiently. The probability distribution of the voxels in the 3D model of Steps 1 and 2 (**M**) is constructed after the candidate patterns 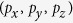 in Step 2 are selected. Then, a “distance” 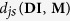, where **DI** represents the digital image, is calculated between the resulting probability distribution of each new 3D model using the candidate pattern 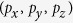. The distance (i.e. the difference) between the two distributions is quantified using the Jensen-Shannon divergence theorem, which is the average of two Kullback-Leibler divergences^25^,^26^,


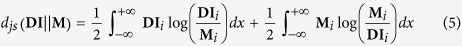


and is always positive. Finally, the central voxel of the pattern that has the minimum 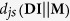 is selected as the final one in **M**. This concept is illustrated in Fig. 9. Conditioning the final 3D model to the probability distribution of the digital image helps one to reproduce both the global structure and the statistical properties. The probability distribution brings both nano- and microscale pores into the final model. Furthermore, the issue of generating a too smooth of a model by using an average as the final pixel value is resolved using this algorithm.

#### Multiresolution properties

One of the most common properties of shales, indicated by high-quality SEM images, is the simultaneous presence of nano- and microscale pore networks that significantly affects the properties of the formation. Current methodologies must be adjusted accordingly to handle very large datasets provided by the SEM images. Thus, a multiresolution approach for addressing such deficiencies as computational cost and pattern reproduction is described.

**Figure 10:**
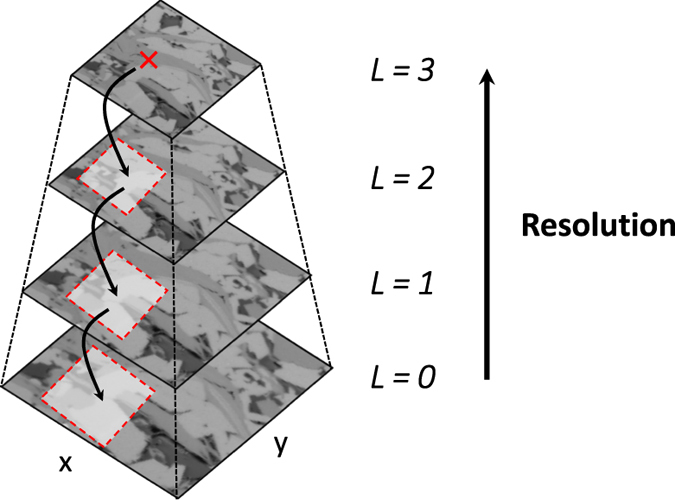
Multiresolution approach for modeling multimodal digital images. The original image is shown at the bottom (*L* = 0). Then, it is upscaled on the basis of the main structures and pore sizes (levels *L* = 0–3). The algorithm begins from the coarsest digital image (*L* = 3) and identifies a matching pattern, the location (red mark) of which is projected onto the next finer digital image (*L* = 2). The new finer digital image is cropped, and only a window around the projected location is searched for the matching pattern. The steps are continued until the matching pattern is identified in the finest-scale digital image, *L* = 0.

The idea is based on a pyramid representation of a large dataset—a digital image in this case. In this method, the original large digital image is up-scaled (coarsened) into successive images with fewer blocks or templates using a nearest-neighbor algorithm; see Fig. 10. The coarsened block that represents *m* × *m* pixels in the finer-scale blocks is assigned the same pixel value as the one among the *m* × *m* pixels with the highest probability. This avoids generating a smooth up-scaled images, most of which fall within the range and distribution of the original digital image due to the nearest-neighbor algorithm. After the images are prepared at various scales (different block sizes), the algorithm is applied as follows: first, the overlap region in the 3D model **G,** reconstructed so far, is resized according to the coarsest available block in the digital image. In other words, if we upscale the digital image into a smaller number of larger blocks by reducing the number of the small blocks by a factor *f*, we do the same with the overlap region. Then, the location of the matching pattern is marked on the coarsest digital image, *L* = 3 in Fig. 10. Next, the identified location is projected onto the finer digital image, and a small window is searched to determine the matching pattern in the finer-scale digital image; see *L* = 2 in Fig. 10. Using the small search window decreases the computational demands tremendously, because (a) it is not necessary to search the entire next finer digital images, and (b) a lower resolution image provides roughly the same information as that of the large digital image. The process is continued until the final resolution, *L* = 0 in Fig. 10, is reached. The selected pattern at this level is inserted directly in the model. It should be noted that the original digital image can be up-scaled until it keeps the same amount of information as the fine-resolution one (i.e. the digital image is not coarsened excessively). In this paper, three levels *L* are used. This multiresolution approach is particularly useful for problems in which the initial digital image is very large and the upscaling does not change enormously the details of the main features. Fortunately, most of currently-available shale images are taken using high-resolution imagining tools and, thus, our technique for reducing the computational burden will be very cost effective.

The multiresolution approach reduces the processing time and preserves the important features effectively. It allows the algorithm to use large templates, thereby producing a better model of large and highly-connected structures, and works well for multimodal shale samples.

#### Mutiscale properties

Depending on their resolution, each of the available imaging tools reveals some information at a specific scale^27^. For example, the widely available 2D/3D low-resolution microtomography images depict large-scale structures. Such images are insufficient for accurate pore space modeling of shales because they cannot display an interconnected pore network. Submicron (nanoscale) images are needed for obtaining a reliable estimation of the permeability and other physical parameters. But, as pointed out earlier, obtaining a large number of high-resolution 2D/3D FIB-SEM images is not affordable. Thus, various images having different resolution and revealing some specific information should be used. Therefore, a methodology that can use the multiscale information and reconstruct a model that contains the statistics of various sources is highly important. The multiscale images depict the final pore network.

Suppose that in addition to the high-resolution digital image used so far, we also have a low-resolution image at larger scale. Then, in addition to the high-resolution 3D model reconstructed so far, we reconstruct a 3D model based on the low-resolution image. Finally, the final multiscale pore network is obtained by overlaying the microscale (high-resolution) and macroscale (low-resolution) 3D models. One can generate many stochastic macroscale 3D models, if the available 3D macroscale image is not representative. It is also worth mentioning that this technique can be used with non-stationary systems even when various datasets need to be integrated^28^,^29^.

## Additional Information

**How to cite this article**: Tahmasebi, P. *et al.* Multiscale and multiresolution modeling of shales and their flow and morphological properties. *Sci. Rep.* **5**, 16373; doi: 10.1038/srep16373 (2015).

## Supplementary Material

Supplementary Information
