# Supplementary material for: Multiscale and multiresolution modeling of shales and their flow and morphological properties
Source: Sci Rep. 2015 Nov 12;5:16373. doi: 10.1038/srep16373 (PMC4642334; doi:10.1038/srep16373)
Supplement: Supplementary Information [file srep16373-s1.pdf]

SUPPLEMENTARY INFORMATION

Multiscale and multiresolution modeling of shales and their flow and morphological properties

Pejman Tahmasebi, Farzam Javadpour, Muhammad Sahimi

Although the method that is described in the paper was used to model shales, it is actually quite general and applicable to modeling of a wide variety of heterogeneous media and materials with a multiscale morphology. To demonstrate this assertion, we have used the method to reconstruct and model two biological materials and an alloy.

The first example is modeling of adipose tissue, loose connective tissue composed mostly of adipocytes. In addition to adipocytes, adipose tissue contains the stromal vascular fraction of cells including preadipocytes, fibroblasts, vascular endothelial cells and a variety of immune cells (i.e., adipose tissue macrophages). Figure S1(a) presents a two-dimensional (2D) sample of such tissues (1). We utilized the method described in the paper to reconstruct the 2D sample. Two realizations are shown in Fig. S1(b). We also reconstructed a 3D model of the tissue, shown in Fig. S1(c). Assuming that the original 3D tissues are isotropic, we computed the multiple-point connectivity probability p(**r**,m) (see Eq. (4) of the paper) for the original 2D sample and the reconstructed 3D model in the horizontal direction. The comparison is shown in Fig. S1(d). The agreement is very good.

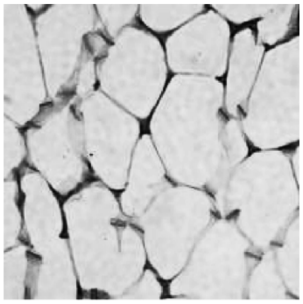

(a)

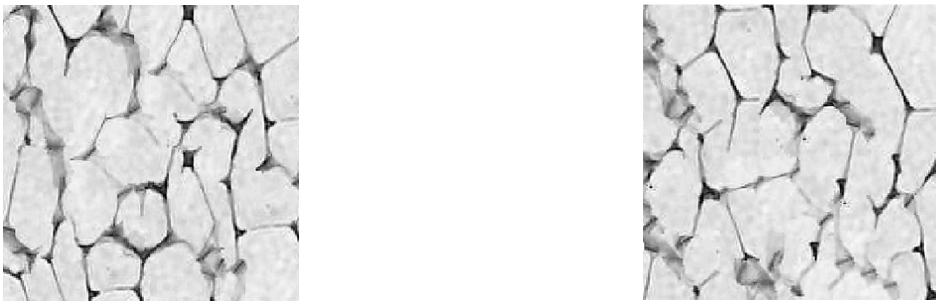

(b)

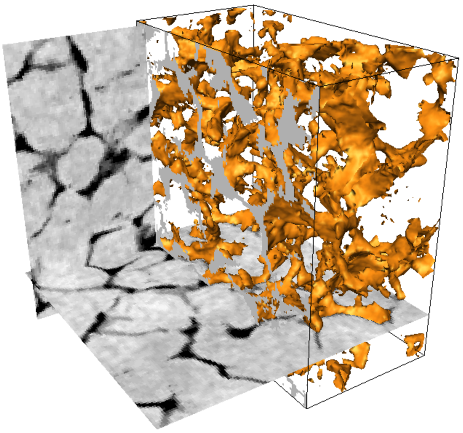

(c)

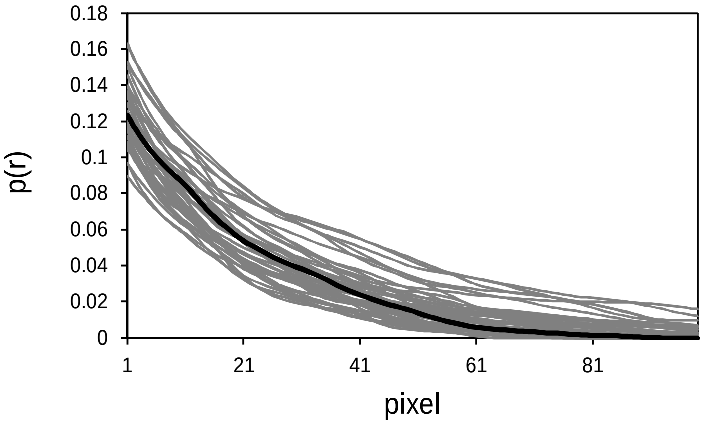

(d)

Figure S1: (a) A sample of adipose tissues. (b) Two realizations of the reconstructed model. (c) A 3D realization of the reconstructed model. (d) Comparison of the multiple-point connectivity probability p(r) of the original 2D sample (black) with that of the reconstructed 3D model (gray) in the horizontal direction.

The second example is a sample of human brain tissue (2). Scientists use stains to color samples of biological tissue before viewing them through light microscopes. Stains highlight particular components of the sample and make it easier to interpret. H&E stain has been used to emphasize the nuclei of the cells (purple). Figure S2 presents the reconstruction.

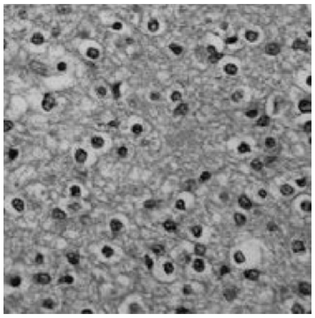

(a)

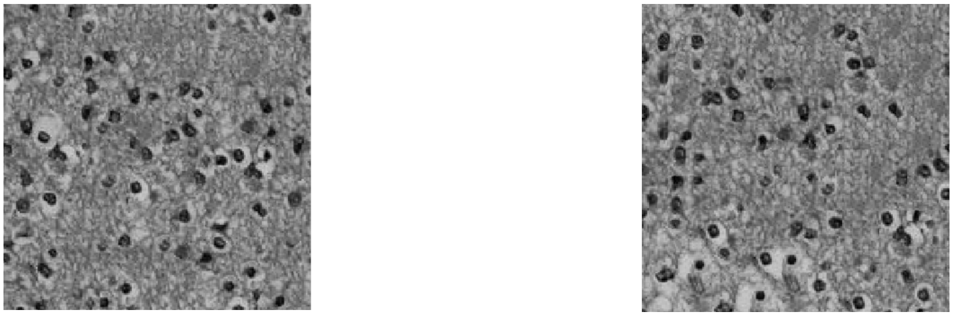

(b)

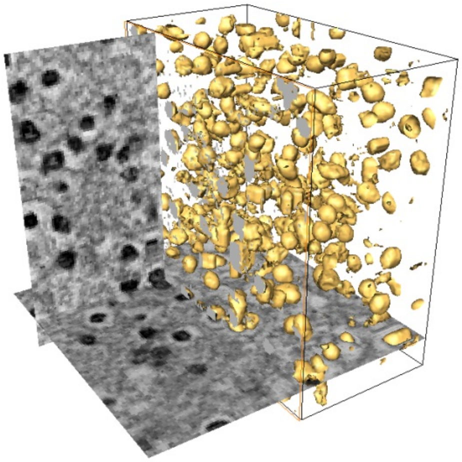

(c)

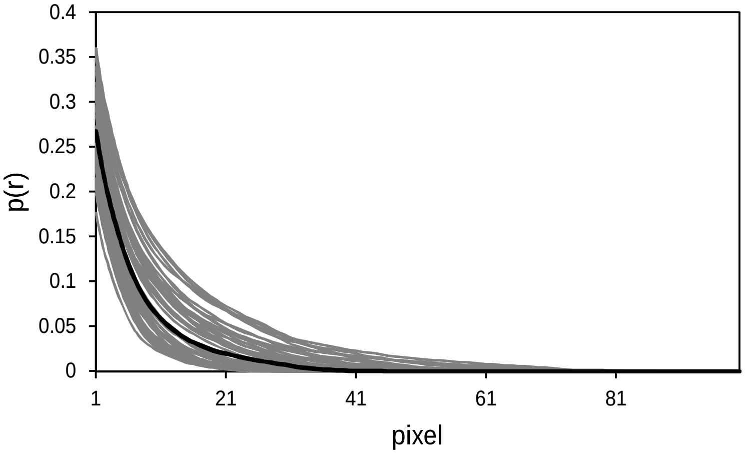

(d)

Figure SI2: Same as in Figure S1, but for a sample of human brain.

The third example (3) represents an image of the microstructure of an alloy, Pd-Ag. The white plates represent the dendritic region, while the black plates represent possibly a large volume of segregated portion.

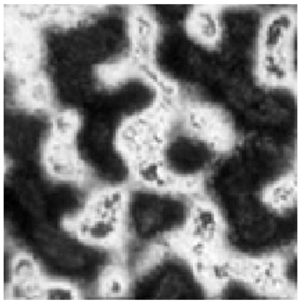

(a)

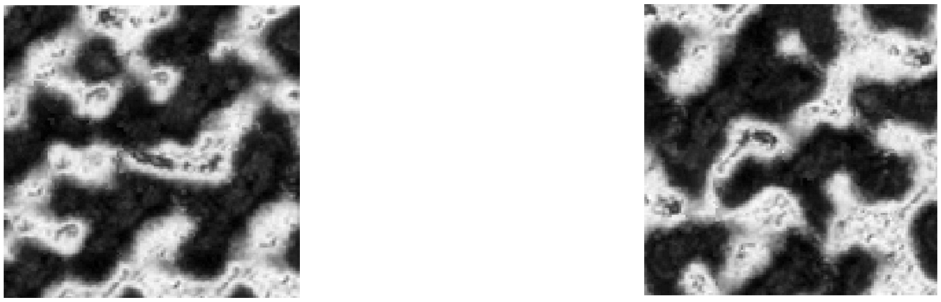

(b)

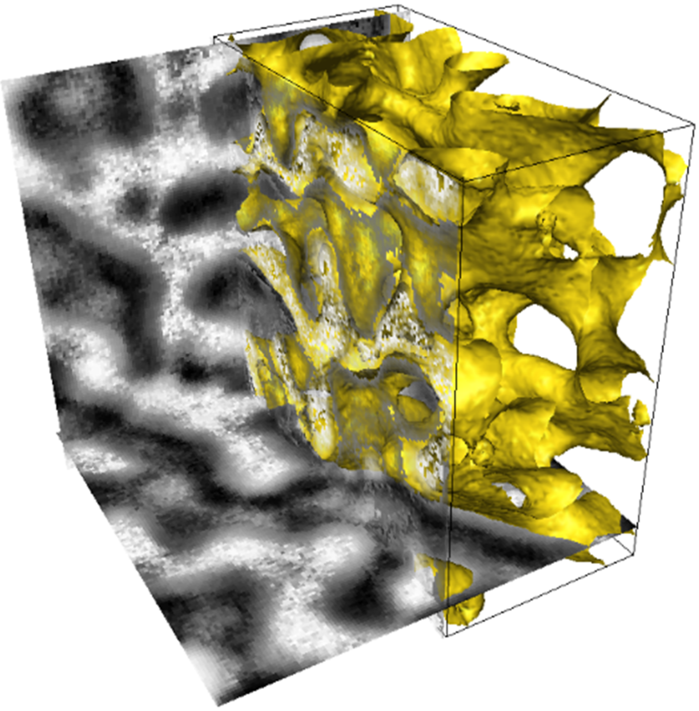

(c)

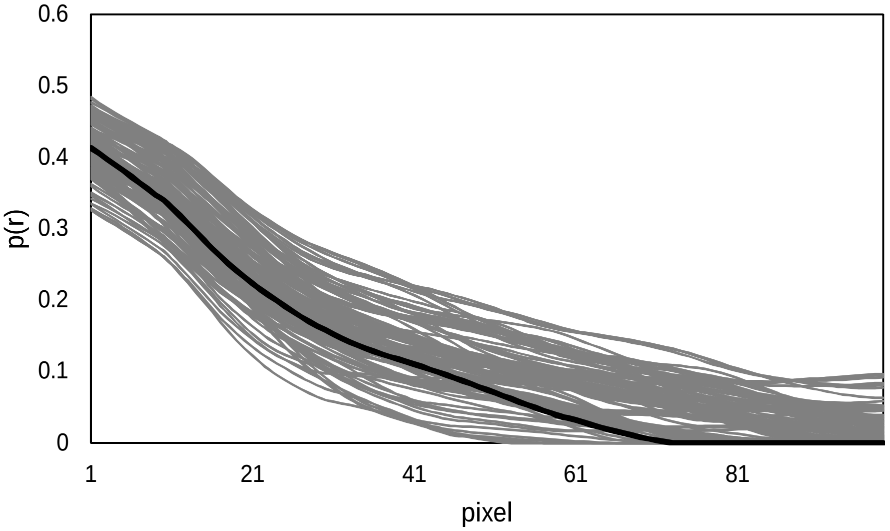

(d)

Figure S3: (a) A sample of Pd-Ag alloy. (b) Two realizations of the reconstructed model. (c) A 3D realization of the reconstructed model. (d) Comparison of the multiple-point connectivity probability p(r) of the original 2D sample (black) with that of the reconstructed 3D model (gray) in the horizontal direction.

References

1. The image was taken from https://www.studyblue.com.
2. The image was taken from http://sciencelearn.org.nz/
3. Bauer, J.R.D.O. et al. Does the casting mode influence microstructure, fracture and properties of different metal ceramic alloys? Braz. Oral Res. **26**, 190-196 (2012).
